# Multi-Level Protective and Risk Factors Longitudinally Associated with Dating Violence Perpetration among Non-Urban Mexican-American Adolescents

**DOI:** 10.3390/adolescents3010005

**Published:** 2022-12-30

**Authors:** Sabrina C. Boyce, Julianna Deardorff, Linda McGlone, Alexandra M. Minnis

**Affiliations:** 1 Center on Gender Equity and Health, School of Medicine, University of California, San Diego, CA 92093, USA; 2 Community Health Sciences, School of Public Health, University of California, Berkeley, CA 94704, USA; 3 Monterey County Health Department, Salinas, CA 93905, USA; 4 Women’s Global Health Imperative, RTI International, San Francisco, CA 94704, USA

**Keywords:** dating violence, Mexican-American, youth, protective factors, family cohesion, bullying

## Abstract

To assess the longitudinal relationship between individual and interpersonal risk and protective factors and dating violence perpetration among non-urban Mexican-American youth. With data from a 24-month prospective cohort study (2015–2019; baseline recruitment spanned from 2015–2017; four follow-up interviews every 6 months) of Mexican-American youth (8th grade at baseline) living in an agricultural region (Salinas, California), we utilized multivariable modified Poisson general estimating equations stratified by gender (*n* = 489) to assess the relationships of religiosity, non-violent problem-solving skills, school connectedness, family cohesion, and bullying victimization with dating violence perpetration. Among girls, but not boys, non-violent problem-solving skills [adjusted relative risk (ARR): 0.7; 95% confidence interval (CI): 0.56–0.99] and family cohesion (ARR: 0.7; 95% CI: 0.48–0.97) were negatively associated with dating violence perpetration, and frequency of bullying victimization was positively associated (ARR: 1.9; 95% CI: 1.37–2.59). Non-urban Mexican-American female youth may benefit from multi-level dating violence prevention that strengthens family cohesion by building upon the Mexican-American cultural value of *familismo* and addresses common risk factors for bullying and dating violence perpetration. Additionally, results affirm etiological differences between girls’ and boys’ dating violence perpetration and the need for improved measurement.

## 1. Introduction

Physical dating violence, estimated to occur in 9% of adolescent girls and 7% of adolescent boys grades 9–11 in the United States [1], has severe psychological, behavioral, and academic consequences across the life-course [2,3]. Psychological dating abuse, often a precursor to physical and sexual violence in later adolescence, can lead to equally deleterious mental and physical health outcomes [2]. Limited research on Hispanic adolescents’ experiences of dating violence (DV) exists, despite evidence indicating that they are at greater risk than non-minority adolescents [1,4]. In California, among urban female youth, who are significantly more likely than their male counterparts to experience dating violence, 62% who report past 12-month physical dating violence are Hispanic and 17% are White [1]. Few DV studies on Hispanic and non-urban youth are available. Recent research, though, emphasizes that youth experience interlocking and mutually constituted experiences of marginalization are at highest risk for physical and sexual dating violence and the most severe consequences of these violent experiences [5]. Non-urban Hispanic youth, who comprise the largest growing racial/ethnic group in non-urban settings across the US, are among these. Rural Hispanic youth have been found to be at similar risk for DV relative to rural White youth (7% vs. 9%, respectively) in early adolescence (grade 6), but then experience elevated risk during late adolescence (grade 12, 23% vs. 12%, respectively) [6], suggesting critical need for early prevention in this population.

Rigorous research on Hispanic youth is needed to inform targeted DV prevention that centers their experiences and culture context, such as risk that arises from discrimination, acculturation (i.e., cross-cultural adaptation and integration), and limited access to social, economic, and health resources in rural settings [7–10]. To date, the majority of DV research on minority youth, including Hispanic and rural youth, has focused on cross-sectional studies and/or DV victimization, rather than more rigorous longitudinal studies of DV perpetration. To better understand DV prevention targets, longitudinal research, which allows for the assessment of temporal ordering of exposures and outcome, is needed to further our understanding of the causal relationship between risk factors and DV. Additionally, research focused on perpetration of DV, the problematic behavior that is central to DV prevention, is essential. Most longitudinal research on perpetration has been conducted among White or mixed-race urban samples with limited focus on cultural or contextual differences [11], which is insufficient to guide prevention for DV perpetration among non-urban Hispanic youth.

### Current Evidence on Interpersonal Targets of Dating Violence Prevention

The Centers for Disease Control and Prevention (CDC) has called for more research to identify prevention targets at the interpersonal and community levels, as individual-level prevention alone is insufficient to reduce DV [12]. Social learning theory of aggression explains violence as a behavior learned from observing and receiving reinforcement from others, particularly from family and peers [13]. Yet, research on interpersonal factors related to DV perpetration is generally lacking, particularly among Hispanic youth. Evidence of interpersonal factors affecting perpetration risk from the limited number of longitudinal studies, primarily among White youth, have identified witnessing parental interpersonal violence and child maltreatment as conferring increased risk [14]. Low school connectedness [15], affiliation with deviant peers [16], and involvement with bullying may also increase risk for perpetration, though findings are mixed on whether experiences with bullying victimization, perpetration or both contribute to risk [16–18]. Additional research that elucidates interpersonal and community factors to target for DV perpetration prevention, specifically among non-urban Hispanic populations, is needed.

Research on protective factors for DV among Hispanic youth is scarce [11]. The traditional focus on risk factors assumes that eliminating risk will promote healthier adolescent dating relationships, yet does not address individual, interpersonal or contextual strengths. Thus, many public health researchers and practitioners have moved toward an asset-based approach, a prevention approach rooted in positive psychology [19] in which factors that protect against a public health problem are promoted. The limited research addressing protective factors suggests that positive parenting strategies, positive peer relationships, and school connectedness [16] may be protective against DV perpetration, yet more research on protective factors is needed to specify what factors should be promoted to help prevent DV among Hispanic youth.

Family cohesion and religion may be important positive interpersonal factors protective against DV perpetration specific to rural Mexican-American populations, as has been found for other health concerns, yet a dearth of research on these relationships exists [20,21]. *Familismo* is a set of normative beliefs within Mexican-American culture that emphasizes the centrality of the family unit, particularly in terms of family obligation, support and emotional closeness, and as a referent for behavioral expectations [22]. Within the cultural context of *familismo*, families that model and expect respectful behavior in relationships may support children to internalize these positive behaviors and then, in turn, express them as non-abusive dating relationship behavior, following social learning theory [13,23]. Previous research, including among Hispanics, has found this effect to be even stronger when the family environment is supportive and emotionally warm, suggesting that taken together with *familismo*, children may be well prepared for healthy interpersonal relationships with future romantic partners, and for rejecting alternative violent models [23,24]. Additionally, research with rural Latinx youth has indicated that family support or closeness may provide protection against mental health symptoms in those that have experienced trauma, likely reducing future risk of violence [25]. Similarly, religiosity among Mexican-American and rural populations, respectively, may also provide protection against DV perpetration by providing positive models of relationships, as well as social support and reinforcement for healthy relationship behavior [26]. Strong family units and religiosity among non-urban Hispanic populations may be assets on which DV perpetration prevention can capitalize.

The current study seeks to build on previous research in critical ways. We aim to provide a longitudinal assessment of risk and protective factors for DV perpetration among a sample of non-urban Mexican-American youth, providing a lens into a population experiencing multiple sources of vulnerability, including structural racism, poverty, isolation, limited access to support resources, and community violence [27]. We examine individual and interpersonal protective factors as well as those that confer risk utilizing data from 24-month follow-up of a prospective longitudinal study of Mexican-American youth living in an agricultural region of California.

## 2. Materials and Methods

### 2.1. Study Setting

The study sample was drawn from youth in Salinas, California, the urban center of an agricultural labor destination for immigrants, predominantly from Mexico, on the Central Coast. Salinas has a vibrant Mexican-American population with rich cultural and social ties, much like many other agricultural regions in California and the Southwest. Youth in Salinas are disproportionally affected by pervasive poverty, exposure to community violence, adolescent pregnancy, and social determinants of health that adversely affect health and wellbeing [28].

### 2.2. Study Design

The A Crecer study is a prospective cohort study of a community sample of Salinas youth (*n* = 599), the design and implementation of which was informed by local youth and parents, a community advisory board, and the local health department [27]. Recruitment took place at the four middle schools that comprise the secondary school district in the study community. The targeted school-based sampling plan was developed in consultation with school leadership and utilized a range of recruitment approaches to capture a diverse sample of students (e.g., classroom announcements, approaching small groups of students in schoolyards). Participants were required to be in 8th grade at baseline attending one of the four middle schools in Salinas, aged 12–15 years, Spanish or English-speaking, provide contact information for a parent to provide permission for participation, and with intent to remain in Salinas over the subsequent year. Study staff obtained verbal parental permission using a structured protocol and written informed assent from participants at enrollment. In-person study visits took place in community-based locations near the middle schools every six months (baseline, 6, 12, 18, and 24-month follow-up). Recruitment of the baseline sample occurred between November 2015–March 2017, a 1.5 year period, and the last of the 24-month follow-up interviews were, therefore, completed in 2019. A 45–60-minute quantitative interview was administered in English or Spanish by an interviewer with sensitive questions administered via audio computer-assisted self-interviewing (ACASI). Participants were re-contacted by study staff (phone calls and text messaging) to schedule follow-up visits, regardless of school retention. Retention of the sample was high (92%) across the study period.

### 2.3. Analytical Sample

We limited the sample for this analysis to male and female-identifying youth reporting dating experience at any time point over the 24-month follow-up. In the full sample, 0.5% of youth identified their sex as non-binary. To protect confidentiality of this very small number of non-binary youth from a small, rural town, they were excluded from the analytic sample. An affirmative response to at least one of the following was considered as having dating experience in the past six months: if they reported having “A boyfriend or girlfriend, or someone you considered a main partner, who you were more serious about than other people?” and/or, “A relationship with someone you wouldn’t call your boyfriend or girlfriend but who was ‘more’ than just a friend? This could be someone you might have gone out with on dates, might have kissed romantically, or who might have been like a ‘friend with benefits’.” The sample was inclusive of youth of all sexual orientations.

### 2.4. Measures

We assessed both individual and interpersonal protective and risk factors hypothesized to be associated with DV perpetration. Selected factors represented potential sources of positive or negative behavioral models that were positively reinforced (e.g., family cohesion, school connectedness, religion, bullying victimization) and non-violent skill development (e.g., non-violent problem-solving skills). These factors were measured every 6 months (all study visits), except for religiosity, which was every 12 months (baseline and 12-month follow-up). School connectedness was assessed using the mean score across eight items (e.g., “I feel like I am part of this school”) with response options ranging from 1–4, 1 indicating “strongly disagree” and 4, “strongly agree” (alpha = 0.79–0.81 at all time points) [29]. Family cohesion, which incorporates the support and emotional closeness aspect of *familismo*, was measured as a mean score across six items (e.g., “Family members feel very close to each other”; response options: 1–4; 4 indicating greater cohesion; alpha = 0.79–0.84) [30]. Bullying victimization was assessed by the question, “How often during the last six months has someone other than a brother or sister done these things to you?” regarding seven forms of bullying (e.g., “Made fun of you for some reason”), a reduced version of the original scale [31]. We utilized a mean of responses (1 = not at all; 2 = once; 3 = more than once) across all seven items (alpha = 0.81–0.82). Non-violent problem-solving skills was assessed as the mean score across four items (e.g., “When I lose my temper, I take my anger out on other people.”; response options: 1–4, 1 indicating “very true” and 4 indicating “not true” (alpha = 0.76–0.82) [32]. Religiosity was assessed as a single item, “How important is religion in your life?” (response options: 1–4, 1 indicating “not at all important” and 4 indicating “very important”) due to limitations in survey length.

The outcome of interest was past six-month DV perpetration assessed at all follow-up visits among participants reporting dating. DV perpetration was defined as an affirmative response to having done any of the following to a dating partner: call them names, insult them, or treat them disrespectfully in front of others; swear at them; threaten with violence or to hurt them; push or shove them; or throw something at them that could hurt them (alpha = 0.76–0.77) [33]. The latter two items were considered physical abuse and the remaining items, psychological abuse. This measure was inclusive of experiences from youth of all sexual orientations and their partners of any gender.

Covariates included age, language acculturation, maternal education, and recruitment school, all assessed at baseline. Language acculturation was measured using a modified version of the Short Acculturation Scale for Hispanic Youth (SASH-Y) scale, a 9-item scale (e.g., “What language(s) do you usually speak in your home?”) assessed using a mean score of response options ranging from 1–5 (1 indicating “only Spanish” and 5, “only English”) [34].

### 2.5. Analysis

Time-lagged independent variables at baseline, 6, 12, and 18 months were utilized to predict perpetration at 6, 12, 18, and 24 months, respectively. Tests for linear trends for descriptive statistics on each panel variable were assessed using linear regression. We utilized bivariable and multivariable general estimating equations with a Poisson distribution, robust standard errors, and exchangeable correlation structures to estimate the longitudinal relationship between independent variables and perpetration stratified by gender, based on previous research that has identified risk and protective factors varying by gender [7,35]. Complete case analyses were conducted; missing data were minimal (<5%). Directed acyclic graphs informed by current research and authors’ expertise were used to identify confounders for each multivariable model. All models adjusted for age and school. Additionally, models for religiosity adjusted for maternal education. Models for non-violent problem-solving skills and school connectedness adjusted for maternal education and language acculturation, and models for bullying victimization adjusted for language acculturation. Analyses were conducted using Stata 15.0 (Stata Corp, LLC, College Station, TX, USA; 2017).

## 3. Results

The sample consisted of 489 male and female identifying youth with dating experience at one or more time points (*n* = 489; 82% of the enrolled sample). They were on average 13 years old at baseline and 42% had a mother with less than high school education (Table 1). The sample consisted of 52% youth reporting their sex to be female, 47% to be male, and 0.5% as non-binary. To protect confidentiality of this very small number of non-binary youth, they were excluded from the analytic sample. On average, participants reported dating in the past 6 months at 3 of the 5 study visits, signaling variation in relationship patterns over time. Across the 24-month follow-up, 28% of participants with dating experience reported perpetrating DV at least once; 30% of female and 25% of male youth reported perpetration. Fewer than 2% reported perpetration at all time points. Across the study period, 26% of participants reported psychological perpetration and 10% reported physical perpetration. No significant linear time trend in perpetration for either sex was observed and differences in DV perpetration by sex were not significant (not shown). Across the 24-month follow-up (from 8th to 10th grade), we observed significant downward trends in levels of school connectedness among male youth (*p* = 0.049) and frequency of bullying victimization among female and male youth (*p* = 0.01 and 0.047, respectively) (Table 2).

Among female youth, non-violent problem-solving skills, family cohesion, and bullying victimization were longitudinally associated with DV perpetration (Table 3). For every additional non-violent problem-solving skill reported, we observed a decrease in risk of perpetration among female youth (adjusted relative risk (ARR): 0.7; 95% confidence interval (CI): 0.56–0.99). At higher levels of family cohesion, female youth’s risk of perpetration was lower (ARR: 0.7; 95% CI: 0.48–0.97). Frequency of reported bullying victimization increased female youth’s risk of perpetration (ARR: 1.9; 95% CI: 1.37–2.59). No statistically significant relationships were observed among male youth. However, the magnitude of the protective effect of non-violent problem-solving skills on DV perpetration was similar to that found for female youth, even though it did not reach statistical significance.

## 4. Discussion

The current study found high levels of past 6-month dating violence among both male and female non-urban Mexican-American youth. Across the 24-month follow-up period, 28% of the sample reported DV perpetration at least once and, on average, 17% of participants reported any perpetration (6% physical and 16% psychological perpetration) at any given follow-up visit. The physical violence estimate is similar to national reports of physical DV victimization [1]. Compared to the perpetration prevalence found in the current study, one study of non-urban Black and White youth estimated a similar prevalence of physical and psychological perpetration among boys (20%) but a lower prevalence for girls (10%) [6]. In our study, despite statistically equivalent levels of perpetration by sex, we identified significant risk and protective factors among female, and not male youth. These findings support previous research that suggests etiological differences in DV perpetration across sex and gender [35]. The high proportion reporting perpetration in this non-urban Mexican-American youth sample underscores the need for early prevention.

This study offers insight into protective factors that may reduce the risk of DV perpetration among female youth. Family cohesion, a construct that reflects the Mexican-American cultural value of *familismo*, provided buffering effects against female perpetration, as hypothesized based on social learning theory. Mexican-American girls may benefit to a greater extent than boys from family cohesion, as previous research has documented Mexican-American girls to have stronger orientation toward family and *familismo* than boys [36]. While only one identified longitudinal study on DV perpetration found significant protective effects for family connection among a Hispanic sample [37], other cross-sectional studies on DV perpetration and studies on DV victimization among Hispanics provide signals that family cohesion is important to perpetration prevention, particularly among girls [7,38]. For Mexican-American youth, promotion of *familismo* may be especially powerful for prevention programming. Few DV prevention interventions address family cohesion; in Salinas, the local health department implements Safe Dates, an evidence-based school-based DV prevention program, in all public high schools in conjunction with parent sessions to improve family communication around healthy relationships. Multi-level prevention that includes both school-based and family-based approaches tailored for Mexican-American families may provide the most promise for promoting *familismo* and healthy relationships among female Mexican-American youth.

Bullying victimization increased risk for subsequent DV perpetration among female youth. The CDC has clarified that multiple forms of violence (e.g., DV, gang violence, bullying) cluster together and should be simultaneously addressed in prevention programming [39]. While most of the research literature focuses on the increased likelihood of DV perpetration among those who have perpetrated bullying, our current findings regarding bullying victimization align with research that has found children who have survived child abuse or witnessed intimate partner violence to be at higher risk for perpetrating violence in future relationships [17,18]. The disempowering experience of being bullied in peer relationships may motivate victimized youth to try to reverse roles and adopt the tactics of those that made them feel inferior in order to feel powerful and regain social status, especially during key adolescent developmental periods in which peer relationships are increasingly important and often involve a strong hierarchical structure [40]. This interpretation is supported by social learning theory that posits that aggressive behavior can be learned via observing or being victim of such behavior and seeing it socially reinforced [13]. Non-violent problem-solving skills have been found to be protective against bullying and DV perpetration [7,14], the latter of which was affirmed for female youth in the current study. As such, programs designed to develop such skills could amplify effects on reduced risk of DV perpetration, both directly and indirectly, via reducing bullying victimization.

Despite high rates of DV perpetration reported by male youth, the current study found no statistically significant factors associated with perpetration among them, affirming other research that has found different correlates of boys’ and girls’ DV perpetration, including among Hispanic youth [7,35]. While many studies, like the current one, have found statistically equivalent levels of DV perpetration among girls and boys [18], evidence shows that boys’ perpetration is more likely to cause injury, emotional distress, and involve sexual violence [7,18,41] and girls’ perpetration against male dating partners is more likely to also involve their own victimization [15]. While not statistically significant at *p* < 0.05, point estimates for non-violent problem-solving skills and bullying victimization among male participants were similar in magnitude to those among female participants, suggesting some importance of these factors for males.

These findings may also signal that there may be some misclassification in the measurement of DV perpetration among male youth; a male youth’s affirmative response to perpetrating DV may have a different meaning than a female youth’s, as severity, the context of power and control and gender norms, and the motivation for using violence (e.g., defensive or instrumental) is not considered. A previous assessment of the modified Conflict Tactics Scale, a shorter version of which was used in the current study, among adolescents in the U.S. demonstrated substantial differences in girls’ and boys’ conceptualization of these aggressive behaviors [42]. Cascardi et al. (1999) suggests that boys may be more likely to deny and minimize psychological and mild physical violence, like the behaviors assessed in this study, while girls may have greater recall of these episodes because they perceive these behaviors as more impactful and fear-inducing [42]. Moreover, current dating violence measures may be more sensitive to capturing girls’ self-defense and/or non-injurious violent behavior and less sensitive to capturing boys’ instrumental, fear-inducing, and injurious violent behavior [41]. Further research to understand differential item functioning in dating violence perpetration measures is needed.

Results should be considered in the context of other limitations. DV perpetration was a self-reported measure subject to self-report bias. Although we utilized ACASI for these sensitive questions to minimize the impact of this bias, it is likely that underreporting still occurred. Religiosity was measured using a single item due to limitations to the length of the survey, but future research may benefit from using a scale that includes a range of items to represent this construct. While no known dating violence interventions were being conducted in the middle schools where youth were recruited, an evidence-based violence prevention program in all local high schools began implementation in Fall 2017 so all youth in the cohort likely were exposed to this program once they transitioned to high school. Future steps with this research will explore effect modification of exposure to high school violence curriculum on significant relationships identified here. Additionally, generalizability of these results to youth who elected not to participate, for whom parent permission to participate was not attainable, or to urban and non-Mexican-American populations is likely limited. Nonetheless, sampling was conducted at all middle schools in the district, 80% of parents for whom contact was attempted were reached, with nearly all (92%) providing permission, and study retention was extremely high (92%), including with participants who discontinued school. Thus, generalizability of findings to this community, as well as to similar Mexican-American agricultural regions in the US, is reasonable.

## 5. Conclusions

Study findings expand the limited research informing prevention on longitudinal protective factors for DV perpetration, providing insight on the unique needs of a growing population experiencing intersecting experiences of DV vulnerability, namely non-urban Mexican-American youth. Female adolescents in this population may benefit from multi-level prevention models that strengthen family cohesion by building upon the salience of the traditional Mexican-American cultural value of *familismo* and addressing risk and protective factors that are shared across DV perpetration and bullying, including teaching non-violent problem-solving skills. The results affirm the importance of considering different etiologies of DV perpetration between boys and girls and suggest the need for careful consideration of possible gender-based bias in the current measurement of DV perpetration in observational and evaluative prevention research.

## Figures and Tables

**Table 1.** Baseline Demographics of Dating Mexican-American Youth in Salinas, CA (*A Crecer, 2015–2019*) (*n* = 489).

	Female (*n* = 253)		Male (*n* = 236)	
Sociodemographics	Mean (SD)	*n* (%)		*n* (%)
Age at baseline	13.24 (0.48)		13.25 (0.53)	
Language acculturation *(1–5; 5 = high acculturation)*	3.44 (0.79)		3.59 (0.83)	
Maternal education:				
Less than HS		114 (45%)		91 (39%)
HS only		75 (30%)		76 (32%)
Post-HS		58 (23%)		60 (25%)
Parent working in agriculture		130 (51%)		111 (47%)
Mexican Origin		220 (87%)		212 (90%)
U.S. generation:				
First generation		31 (12%)		28 (12%)
Second generation		181 (72%)		165 (70%)
Third or later generation		38 (15%)		41 (17%)
Number of visits (1–5) at which dating was reported	3.24 (1.47)		3.20 (1.45)	

Abbreviation: SD, standard deviation; HS, high school.

**Table 2.** Time-varying Longitudinal Independent and Dependent Variables of Interest among Dating Mexican-American Youth in Salinas, Ca (A Crecer, all time points, 2015–2019) (n = 489).

	Female (*n* = 253)						Male (*n* = 236)					
	0 m (*n* = 171)	6 m (*n* = 147)	12 m (*n* = 162)	18 m (*n* = 169)	24 m (*n* = 171)	*p*-value^a^	0 m (*n* = 196)	6 m (*n* = 149)	12 m (*n* = 132)	18 m (*n* = 134)	24 m (*n* = 144)	*p*-value^a^
	*mean (SD)*						*mean (SD)*					
Individual factors:												
Religiosity *(1–4; 4 = most religious)*	3.21	.	3.01	.	.	0.06	3.09	.	2.95	.	.	0.33
	(0.74)	.	(0.74)	.	.		(0.80)	.	(0.84)	.	.	
Non-Violent Problem-Solving Skills *(1–4; 4 = most skilled)*	2.48	2.48	2.60	2.62	.	0.51	2.92	2.98	2.81	3.04	.	0.51
	(0.69)	(0.79)	(0.80)	(0.82)	.		(0.74)	(0.83)	(0.88)	(0.83)	.	
Interpersonal factors:												
School connectedness *(1–4; 4 = high belonging)*	3.19	3.18	3.14	3.13	.	0.07	3.31	3.29	3.28	3.18	.	0.05
	(0.44)	(0.42)	(0.42)	(0.36)	.		(0.42)	(0.40)	(0.43)	(0.45)	.	
Family cohesion *(1–4; 4 = very close)*	3.21	3.12	3.14	3.14	.	0.09	3.33	3.29	3.21	3.23	.	0.52
	(0.45)	(0.52)	(0.49)	(0.50)	.		(0.45)	(0.47)	(0.57)	(0.55)	.	
Bullying victimization *(1–3; 1 = never; 3 = more than once)*	1.68	1.51	1.45	1.40		0.01	1.50	1.36	1.34	1.32	.	0.05
	(0.48)	(0.47)	(0.49)	(0.49)	.		(0.47)	(0.46)	(0.46)	(0.41)	.	
	*n (%)*						*n (%)*					
Dating violence												
Perpetration	.	33 (23%)	24 (15%)	28 (17%)	37 (22%)	0.97	.	29 (19%)	21 (16%)	15 (11%)	21 (15%)	0.26
Verbal	.	30 (20%)	21 (13%)	25 (15%)	34 (20%)		.	25 (17%)	21 (16%)	13 (10%)	21 (15%)	
Physical	.	10 (7%)	6 (4%)	10 (6%)	15 (9%)		.	9 (6%)	6 (5%)	4 (3%)	5 (4%)	

Abbreviation: SD, standard deviation,

a p-value for linear test for trend (linear regression), Missing statistics indicates that at this data collection time point, these data were not collected and/or included in the analysis.

**Table 3.** Longitudinal Unadjusted and Adjusted Relative Risks of Dating Violence Perpetration among Dating Mexican-American Youth in Salinas, CA (*A Crecer*, all time points, 2015–2019) (*n* = 489).

	Female (*n* = 253)				Male (*n* = 236)			
	DVP	No DVP	Relative Risk^a^		DVP	No DVP	Relative Risk^a^	
	mean (SD)*	mean (SD)*	Unadjusted 95% CI	Adjusted 95% CI	mean (SD)*	mean (SD)*	Unadjusted 95% CI	Adjusted 95% CI
**Individual factors**								
Religiosity^b^	2.98 (0.83)	3.07 (0.72)	0.8 (0.64–1.06)	0.8 (0.64–1.07)	3.01 (0.91)	3.01 (0.83)	1.0 (0.76–1.33)	1.0 (0.76–1.32)
Non-violent problem-solving skills^c^	2.36 (0.77)	2.62 (0.76)	0.7 (0.56–0.98)	0.7 (0.56–0.99)	2.69 (0.94)	2.94 (0.78)	0.8 (0.61–1.05)	0.8 (0.60–1.05)
**Interpersonal factors**								
School connectedness^c^	3.16 (0.39)	3.17 (0.42)	1.1 (0.72–1.56)	1.1 (0.72–1.56)	3.25 (0.40)	3.27 (0.43)	1.0 (0.63–1.64)	1.0 (0.60–1.55)
Family cohesion^d^	3.08 (0.53)	3.19 (0.48)	0.7 (0.48–1.00)	0.7 (0.48–0.97)	3.24 (0.53)	3.29 (0.49)	0.9 (0.59–1.41)	0.9 (0.60–1.43)
Bullying victimization^d^	1.71 (0.54)	1.46 (0.46)	1.9 (1.37–2.58)	1.9 (1.37–2.59)	1.50 (0.51)	1.36 (0.42)	1.5 (0.89–2.40)	1.4 (0.87–2.39)

Abbreviations: DVP, dating violence perpetration; SD, standard deviation; CI, confidence interval

* mean and standard deviation (SD) across all time points

a Unadjusted and adjusted relative risks were estimated using generalized estimating equations with robust standard errors, exchangeable correlation structure, and Poisson distribution

b Adjusted model for age, maternal education, site

c Adjusted model for age, maternal education, language acculturation score, site

d Adjusted model for age, language acculturation score, site.

## Data Availability

The data presented in this study are available on request from the corresponding author. The data are not publicly available due to privacy and ethical restrictions.
